# POEM Is a Durable Treatment in Children and Adolescents With Achalasia Cardia

**DOI:** 10.3389/fped.2022.812201

**Published:** 2022-02-25

**Authors:** Zaheer Nabi, Mohan Ramchandani, Jahangeer Basha, Rajesh Goud, Santosh Darisetty, Duvvur Nageshwar Reddy

**Affiliations:** Department of Gastroenterology, Asian Institute of Gastroenterology, Hyderabad, India

**Keywords:** achalasia, child, adolescents, per-oral endoscopic myotomy, outcome (recurrence, chronicity)

## Abstract

**Background and Aim:**

Per-oral endoscopic myotomy (POEM) is emerging as an effective treatment for pediatric achalasia. There are limited data on the long-term efficacy of POEM in children and adolescents with achalasia. In this study, we aim to evaluate the outcomes of POEM at ≥4 years follow-up.

**Method:**

The data of consecutive children who underwent POEM (September 2013–July 2021) and completed at least 4 years follow-up were analyzed retrospectively. The primary outcome was clinical success (Eckardt ≤ 3) at ≥4 years follow-up. The secondary outcomes included the prevalence of gastroesophageal reflux disease (GERD) and predictors of recurrent symptoms (Eckardt ≥2) after POEM.

**Results:**

A total of 69 children underwent POEM for achalasia during the study period. Of these, 41 (59.4%) children completed ≥4 years [mean 68.5 months (range 48–94)] follow-up, and 38 were included in the final analysis. The subtypes of achalasia included type I (28.9%), type II (60.5%), and type III (2.6%). There was a history of prior treatment in 11 children (28.9%). Clinical success was recorded in 36 (94.7%) patients who successfully underwent POEM. Recurrent symptoms (Eckardt ≥ 2) were noticed in 12 (31.6%) children at ≥4 years. On multivariate analysis, there were no identifiable factors which predicted recurrent symptoms after POEM. Symptomatic GERD and erosive esophagitis were detected in 13.8% (4/29) and 57.1% (8/14) of the children, respectively.

**Conclusion:**

POEM is a durable treatment modality for achalasia in the pediatric population irrespective of the sub-type of achalasia and history of prior treatment.

## Introduction

Achalasia cardia is rare in the pediatric population, and <5% of all cases present below 15 years of age. The major modalities for the management of achalasia include pneumatic dilatation and Heller's myotomy. More recently, per-oral endoscopic myotomy (POEM) has been introduced as an endoscopic treatment option for achalasia. Multiple studies have established the safety and efficacy of POEM in adults with achalasia. Emerging data suggest that POEM may be an effective treatment in children and adolescents as well (1–10). However, there are limited data on the long-term outcomes of POEM in the pediatric population.

In this study, we aimed to analyze the long-term outcomes of POEM in children and adolescents with achalasia.

## Methods

The data of children and adolescents (age ≤ 19 years) who underwent POEM for achalasia from September 2013 to July 2021 were analyzed retrospectively. Pediatric cases who completed at least 4 years of follow-up were included in the study. The study was approved by the institutional review board committee (AIG/AHF IRB: 34/2015).

The inclusion criteria were as follows:

a) Treatment naïve or previously treated cases with achalasia
b) Age ≤ 19 years
c) Minimum follow-up of 4 years

The exclusion driteria were as follows:

a) Follow-up <4 years
b) Refusal to provide written informed consent

### Pre-POEM Evaluation

The diagnosis of achalasia was established using high-resolution esophageal manometry (HREM), timed barium esophagogram, and upper gastrointestinal endoscopy. The technique of manometry in children has been described in our previous study (7). The type of esophageal motility disorder and lower esophageal sphincter (LES) pressures were recorded on manometry.

### POEM Technique

**Figure 1:**
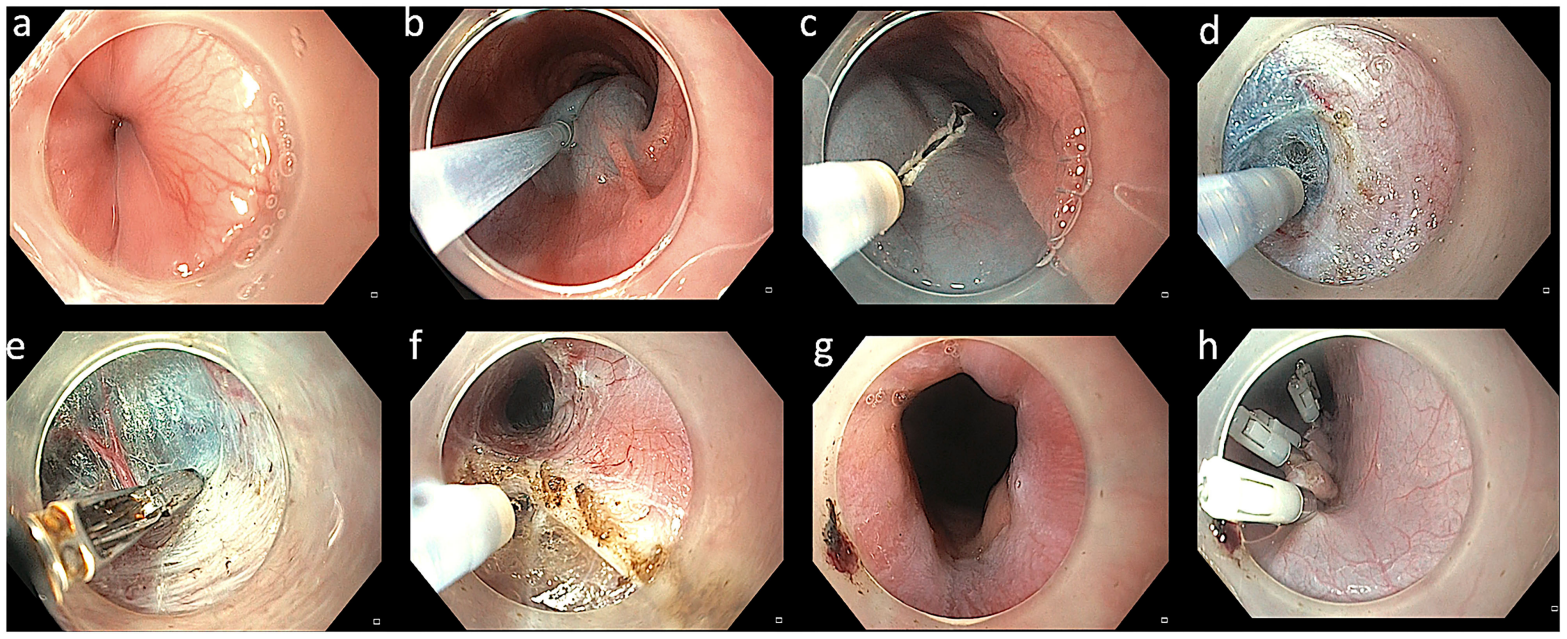
Technique of per-oral endoscopic myotomy in a case with achalasia. **(a)** Endoscopy revealing a tight gastroesophageal junction. **(b)** Mucosal lifting injection with diluted indigocarmine dye. **(c)** Mucosal incision using triangular tip knife. **(d)** Submucosal dissection using triangular knife. **(e)** Coagulation of vessels using coagulation forceps. **(f)** Myotomy using triangular knife. **(g)** Wide open gastroesophageal junction after completion of myotomy. **(h)** Closure of mucosal incision using multiple endoclips.

All the POEM procedures were performed by three operators (MR, ZN, and DNR) by anterior or posterior route using standard technique previously described (Figure 1) (11). Posterior myotomy was preferred in cases with a history of Heller's myotomy. Post-procedure, oral contrast study was performed on the second post-operative day before initiating oral diet.

### Follow-Up Protocol

All patients were followed at pre-defined intervals, i.e., 3 months, 1 year, and annually thereafter. Evaluation at each visit included symptom assessment for achalasia (Eckardt score) as well as gastroesophageal reflux (heartburn and regurgitation). Objective evaluation including esophageal manometry and 24-h pH impedance study were performed at 3 months after POEM.

### Primary Outcome

The primary outcome of the study was clinical efficacy at ≥4 years follow-up. Clinical success was defined using Eckardt score which is a composite score consisting of sub-scores for dysphagia (0–3), regurgitation (0–3), chest pain (0–3), and weight loss (0–3). The minimum and maximum possible scores are 0 and 12, respectively. Clinical success was defined as Eckardt score ≤ 3. The outcomes were recorded during annual follow-up visits. All the patients who completed ≥4 years and could not come for physical visits were contacted by telephonic questionnaire for clinical success and symptomatic gastroesophageal reflux disease (GERD). Clinical failure was defined as Eckardt score >3. Recurrence of symptoms was defined as any degree of symptoms but not amounting to clinical failure (Eckardt >1 and ≤ 3).

### Secondary Outcomes

The secondary outcomes included the predictors of recurrent symptoms (Eckardt ≥ 2) after POEM. Reflux esophagitis was graded according to the Los Angeles classification system (LA grades A to D) (12).

## Statistics

The continuous data were expressed as mean (standard deviation) or median (interquartile range) and compared with independent sample *t*-test and the categorical data as frequencies and compared with chi-square test unless otherwise specified. The comparison of Eckardt score between pre- and post-POEM (at 1 and 4 years) was done using repeated measure analysis of variance (ANOVA). Multivariate analysis was performed using binominal logistic regression to ascertain the effects of age, gender, type of achalasia, Eckardt score, and LES pressures (pre- and post-POEM) on recurrence of symptoms at long-term follow-up. The linearity of the continuous variables with respect to the logit of the dependent variable was assessed via the Box–Tidwell procedure. All the tests of significance were two-tailed, and a *p* < 0.05 was considered to indicate statistical significance.

## Results

A total of 69 children underwent POEM for achalasia during the study period. Of these, 41 (59.4%) children completed ≥4 years [mean 68.5 months (range 48–94)] follow-up. Data on clinical efficacy were available in 38 children and were included in the final analysis. The spectrum of motility disorders included 11 (28.9%) with type I achalasia, 23 (60.5%) with type II achalasia, and 1 (2.6%) with type III achalasia. Manometry data were not available in three children. A history of prior treatment was present in 11 (28.9%; Table 1).

**Table 1:** Baseline characteristics of per-oral endoscopic myotomy in children.

Variable	38
Age in years, mean (SD)	14.7 ± 3.3 (range 4–19)
Gender, M/F	23/15
Type of achalasia (I/II/III)^a^	11/23/1
Pre-POEM Eckardt score, median (IQR)	7 (6–8)
Pre-POEM LES pressure, mean (SD)	35.4 ± 12.5
Prior treatment	11 (28.9%) BD (1): 5; BD (>1): 4; Heller's myotomy: 1; BD and Heller's: 1
POEM procedure (technical details)	
Orientation of myotomy: Anterior (%):	32 (84.2)
Posterior (%)	6 (15.8)
Esophageal myotomy in cm, mean (SD)	7.8 ± 2.2
Gastric myotomy in cm, mean (SD)	3.0 ± 0.6
Procedure duration in minutes, median (IQR)	50 (40–100)

a *Manometry details not available in three children*.

*SD, standard deviation; POEM, per-oral endoscopic myotomy; IQR, interquartile range; LES, lower esophageal sphincter; BD, balloon dilatation*.

POEM was technically successful in 37 (97.4%) children. POEM was deferred in one child due to the child's small size and neurological problems. Baseline Eckardt score, manometry parameters, and intra-operative details including length and orientation of myotomy have been outlined in Table 1.

### Primary Outcome

**Figure 2:**
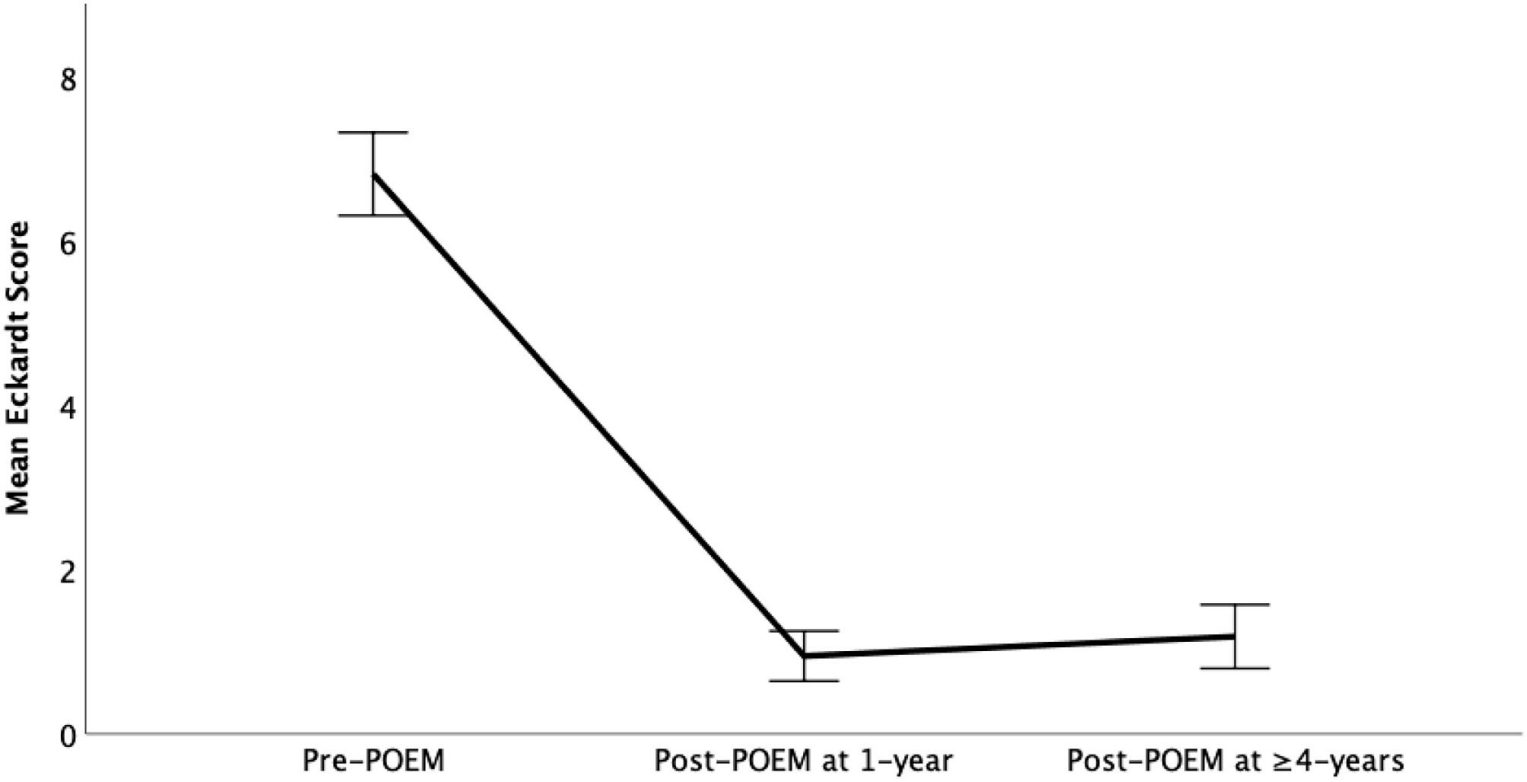
Comparison of mean Eckardt scores before and after per-oral endoscopic myotomy.

The data regarding efficacy was available in 38/41 (92.7%) patients. Clinical success was recorded in 36 (94.7%) patients who successfully underwent POEM and were available for final follow-up. In intention to treat analysis [including technical failures (1, 2.6%) and lost to follow-up (2, 7.9%)], the clinical success in the overall group was observed in 36/42 (85.7%). There was significant reduction in the median Eckardt scores at 1 year [1(0–1)] and ≥4 years [1(0–2)] as compared to baseline [7(6–8)] (*p* < 0.001; Figure 2 and Table 2).

**Table 2:** Long-term outcomes of per-oral endoscopic myotomy (≥48 months).

	**Number = 38**
Clinical success, *n* (%)	36 (94.7)
Eckardt score, median (IQR)	1 (0–2)
**Post-POEM reflux**	
Reflux esophagitis (*n* = 14)	
Grade A, *n* (%)	6 (42.8)
Grade B, *n* (%)	2 (14.3)
Symptoms of GERD (*n* = 29)	
Heartburn and regurgitation, *n* (%)	4 (13.8)
Follow-up in months, mean (SD)	68.5 (16.1)

*IQR, interquartile range; POEM, per-oral endoscopic myotomy; GERD, gastroesophageal reflux disease; SD, standard deviation*.

### Secondary Outcome

The secondary outcomes included predictors of recurrent symptoms and incidence of GERD after POEM.

### Recurrent Symptoms

Recurrent symptoms equivalent to Eckardt ≥2 were noticed in 12 (31.6%) children at ≥4 years. On univariate and multivariate analysis, factors including baseline Eckardt score, type of achalasia, length and orientation of myotomy, baseline LES pressure, and history of prior treatment had no significant impact on the recurrence of symptoms after POEM (Table 3).

**Table 3:** Multivariate analysis for prediction of recurrent symptoms after per-oral endoscopic myotomy.

**Variable**	**Eckardt ≤ 1 (*n* = 26)**	**Eckardt ≥ 2 (*n* = 12)**	***p***	**Adjusted odds ratio (95% CI)**	***p***
Gender, M (%)	17 (65.4)	6 (50)	0.481	3.41 (0.30–38.26)	0.320
Mean age (SD)	15.1 (3.5)	13.9 (2.8)	0.321	0.85 (0.51–1.41)	0.531
Prior treatment (%)	5 (19.2)	6 (50)	0.068	0.14 (0.01–1.98)	0.146
Type of achalasia (II vs. Others)	16 (61.5)	7 (58.3)	0.897	–	0.562
Mean Eckardt (SD)	6.8 (1.5)	6.7 (1.6)	0.861	1.06 (0.50–2.22)	0.886
Anterior POEM	23 (71.9)	9 (28.1)	0.357	0.61 (0.01–86.06)	0.845
Mean esophageal myotomy (SD)	7.9 (2.4)	7.4 (1.9)	0.489	1.13 (0.55–2.31)	0.745
Mean gastric myotomy (SD)	2.9 (0.6)	3.2 (0.7)	0.262	9.26 (0.54–157.50)	0.124
Pre-LES pressure, mean (SD)	37.3 (12.7)	31.2 (11.5)	0.202	0.92 (0.80–1.07)	0.303
Post-LES pressure, mean (SD)	12.2 (5.6)	11.5 (3.8)	0.711	1.07 (0.68–1.68)	0.760

*SD, standard deviation; POEM, per-oral endoscopic myotomy; LES, lower esophageal sphincter*.

### Gastroesophageal Reflux Disease

The data on symptomatic GERD and reflux esophagitis was available in 29 (76.3%) and 14 (36.8%) children, respectively. Symptoms of GERD were evident in 4 (13.8%) children. Erosive esophagitis was detected in 8 (57.1%). All cases had mild (LA grade A: 6 and B: 2) esophagitis (Table 2).

## Discussion

In this study, we found POEM to be an effective and durable treatment modality in children and adolescents with achalasia cardia. Although erosive esophagitis was detected in over half of the children, severe esophagitis and symptomatic GERD were uncommon on long-term follow-up.

The safety and short-term efficacy of POEM has been established in adult patients with achalasia as well as non-achalasia spastic motility disorders of the esophagus. In pediatric cases with achalasia, Heller's myotomy and pneumatic dilatation are the preferred treatment modalities (13). Emerging data suggest that POEM is an effective alternative to pneumatic dilatation and Heller's myotomy in pediatric achalasia as well (6–8). However, there is limited data to suggest the long-term efficacy of POEM in children and adolescents. Since achalasia is a progressive disease, long-term outcomes are crucial to establish the durability of POEM in esophageal achalasia.

In this study, we evaluated the outcomes of POEM including clinical success and GERD in pediatric cases who completed at least 4 years follow-up. The mean follow-up of the entire cohort was 69 months, i.e., >5 years. Overall, clinical success was recorded in 95% (per protocol) and 86% (intention to treat) of children at ≥4 years follow-up. In previously published studies with relatively long follow-up periods, the clinical success rate of POEM in pediatric achalasia ranged from 95 to 100% at follow-up ranging from 26 to 40 months (3, 8, 14, 15) (Table 4). Our results suggest that in pediatric achalasia, the response to POEM is sustained for at least 4 years. The results are similar to those in adult patients in whom clinical success has been recorded in 80–95% of cases at a median follow-up ranging from 3 to 7 years (16–25).

**Table 4:** Selected studies depicting the outcomes of POEM in children at ≥2 years follow-up.

**References**	***N***	**Age, years Mean (SD or range)**	**Prior treatment**	**Clinical success (%)**	**Follow-up, mean (SD)/median (range)**
Tan et al. (3)	12	13.7 (2.6)	NR	100	26 m
Mangiola et al. (14)	26	10.9 (2–17)	2 (BD)	100	30.6 m (15)
Yamashita et al. (15)	7	15 (3.1)	NR	100	39.6 m (18–54)
Liu et al. (8)	130	NR	20 (BD 12, stent 3, BTX 1, HM 3, POEM 1)	95.6	40 m (4–88)
Current study	38	14.7 ± 3.3 (4–19)	11 BD (1): 5; BD (>1): 4; HM: 1; BD and HM: 1	94.7	68.5 m (16.1)

*SD, standard deviation; NR, not reported; BD, balloon dilatation; BTX, botulinum toxin injection; HM; Heller's myotomy; POEM, per-oral endoscopic myotomy*.

Although there is no randomized trial in children, the clinical success rates with POEM appear to be higher as compared to Heller's myotomy and pneumatic dilatation (26, 27). In a recent systematic review, the clinical success with Heller's myotomy was 78% at a mean follow-up of 3 years and 45% with pneumatic dilatation at an average follow-up of 3.5 years (27).

There were only two clinical failures on long-term follow-up. Consequently, we analyzed the risk factors for recurrent symptoms equivalent to Eckardt >1 on long-term follow-up. A majority of the cases with recurrent symptoms had occasional dysphagia and or regurgitation (equivalent to Eckardt score of ≤2). On multivariate analysis, there were no predictors for recurrent symptoms at long-term follow-up. However, it is important to note that the analysis was intended for the prediction of recurrent symptoms and not clinical failure. Large, multicenter studies are required to establish the predictors of outcomes and optimize the use of POEM in pediatric achalasia.

Symptoms of GERD and erosive esophagitis were detected in 14% and 57% of children, respectively. GERD is a significant issue after POEM and more frequent as compared to pneumatic dilatation and Heller's myotomy with fundoplication (28). In adults, GERD is evident in almost half of the patients on 24-h pH study, and reflux esophagitis is noted in 20–40% of patients after POEM (29–31). In this study, all the children had mild reflux esophagitis (≤LA grade B) suggesting that GERD may not be a major hindrance while adopting POEM in the management algorithm for pediatric cases. Our results are in concordance with a recent study in adult patients in which a majority of the patients were asymptomatic for GERD, developed mild esophagitis (Los Angeles grade A or B), and responded well to proton pump inhibitor therapy (31).

We acknowledge that objective evaluation of GERD could not be performed in a substantial proportion of cases on long-term follow-up. Therefore, the possibility of selection bias cannot be completely ruled out. In addition, the symptoms of GERD like regurgitation, heartburn, and chest pain can closely mimic those of achalasia which may confound the interpretation of results. Nevertheless, our study provides some reassurance regarding this potential long-term complication of POEM.

There are several strengths of our study. To the best of our knowledge, this is one of the largest studies evaluating the long-term outcomes of POEM (≥4 years). The number of cases who were lost to follow-up were within an acceptable range (<10%). We acknowledge a few noteworthy limitations of this study. These include the retrospective design and lack of objective evaluation of success and GERD using timed barium swallow, esophageal manometry, and endoscopy. The evaluation of symptoms was based on Eckardt score, which has not been validated in pediatric patients. Similarly, there is limited information regarding the interpretation of esophageal manometry in children. The impact of POEM on the nutritional status of the children could not be assessed due to incomplete information.

## Conclusion

POEM is a durable treatment option for achalasia cardia. There is no substantial impact of the sub-type of achalasia and prior treatment on the long-term outcomes of POEM.

## Data Availability Statement

The raw data supporting the conclusions of this article will be made available by the authors, without undue reservation.

## Ethics Statement

The studies involving human participants were reviewed and approved by AIG/AHF IRB. Written informed consent to participate in this study was provided by the participants' legal guardian/next of kin.

## Author Contributions

ZN and MR were involved in the conception of the study. JB and RG were involved in acquisition and analysis of the data. SD and DR were involved in revising the manuscript for important intellectual content. All authors agreed to the final version of the manuscript.

## Conflict of Interest

The authors declare that the research was conducted in the absence of any commercial or financial relationships that could be construed as a potential conflict of interest.

## Publisher's Note

All claims expressed in this article are solely those of the authors and do not necessarily represent those of their affiliated organizations, or those of the publisher, the editors and the reviewers. Any product that may be evaluated in this article, or claim that may be made by its manufacturer, is not guaranteed or endorsed by the publisher.
